# Microbiomic Analysis of Bacteria Associated with Rock Tripe Lichens in Continental and Maritime Antarctic Regions

**DOI:** 10.3390/jof8080817

**Published:** 2022-08-03

**Authors:** Zichen He, Takeshi Naganuma, Ryosuke Nakai, Satoshi Imura, Megumu Tsujimoto, Peter Convey

**Affiliations:** 1 Graduate School of Integrated Science for Life, Hiroshima University, 1-4-4 Kagamiyama, Higashi-Hiroshima 739-8528, Japan; he-zichen@hiroshima-u.ac.jp; 2 Bioproduction Research Institute, National Institute of Advanced Industrial Science and Technology, 2-17-2-1 Tsukisamu-Higashi, Sapporo 062-8517, Japan; nakai-ryosuke@aist.go.jp; 3 National Institute of Polar Research, 10-3 Midori-Cho, Tachikawa 190-8518, Japan; imura@nipr.ac.jp (S.I.); megumu@sfc.keio.ac.jp (M.T.); 4 Department of Polar Science, SOKENDAI (The Graduate University for Advanced Studies), 10-3 Midori-cho, Tachikawa 190-8518, Japan; 5 Faculty of Environment and Information Studies, Keio University, 5322 Endo, Fujisawa 252-0882, Japan; 6 British Antarctic Survey, High Cross, Madingley Road, Cambridge CB3 0ET, UK; pcon@bas.ac.uk; 7 Department of Zoology, University of Johannesburg, Auckland Park, Johannesburg 2006, South Africa; 8 Millennium Institute Biodiversity of Antarctic and Subantarctic Ecosystems (BASE), Santiago 7800003, Chile; 9 Cape Horn International Center (CHIC), Puerto Williams 6350000, Chile

**Keywords:** *Umbilicaria*, Antarctica, rRNA gene, V3-V4 region, MiSeq, OTUs, biogeography, metabolism

## Abstract

Increased research attention is being given to bacterial diversity associated with lichens. Rock tripe lichens (*Umbilicariaceae*) were collected from two distinct Antarctic biological regions, the continental region near the Japanese Antarctic station (Syowa Station) and the maritime Antarctic South Orkney Islands (Signy Island), in order to compare their bacterial floras and potential metabolism. Bulk DNA extracted from the lichen samples was used to amplify the 18S rRNA gene and the V3-V4 region of the 16S rRNA gene, whose amplicons were Sanger- and MiSeq-sequenced, respectively. The fungal and algal partners represented members of the ascomycete genus *Umbilicaria* and the green algal genus *Trebouxia*, based on 18S rRNA gene sequences. The V3-V4 sequences were grouped into operational taxonomic units (OTUs), which were assigned to eight bacterial phyla, *Acidobacteriota*, *Actinomyceota*, *Armatimonadota*, *Bacteroidota*, *Cyanobacteria*, *Deinococcota*, *Pseudomonadota* and the candidate phylum Saccharibacteria (also known as TM7), commonly present in all samples. The OTU floras of the two biological regions were clearly distinct, with regional biomarker genera, such as *Mucilaginibacter* and *Gluconacetobacter*, respectively. The OTU-based metabolism analysis predicted higher membrane transport activities in the maritime Antarctic OTUs, probably influenced by the sampling area’s warmer maritime climatic setting.

## 1. Introduction

Lichens are common and widely distributed symbiotic organisms. Although they are not charismatic in appearance, they occur in a wide variety of habitats and environmental conditions. While lichens photosynthesize and may superficially resemble mosses and grow with them, they are not phylogenetically related to mosses or other plant groups [1,2]. When lichens are present epiphytically, such as on tree trunks and branches or on moss surfaces, they occur commensally using the plants as colonization substrates. The physical structure of lichens is provided by lichen-forming fungi [3], which provide the formal scientific name of the lichen, while the photosynthetic function is provided by symbiotic microalgae and/or cyanobacteria [4,5,6], termed the mycobiont and photobiont, respectively. The entire lichen is a symbiotic holobiont consisting of a fungal and one or more algal/cyanobacterial partners.

Lichens require air, water, micronutrients and substrates to survive [7]. Like mosses, they cannot regulate their hydrological balance. However, as a group, they show impressive cryptobiotic adaptations and can tolerate and grow under conditions of irregular water supply, as well as showing high tolerance to severe abiotic conditions, such as extreme temperatures and high levels of light and ultraviolet radiation [8,9]. Based on these adaptations, lichens are often the pioneer microorganisms colonizing extreme environments such as montane areas, hot and cold deserts and the polar regions.

In addition to the symbiotic partnership between fungal and algal/cyanobacterial bionts, bacteria have also attracted increasing research attention in recent years as a third biotic component present within lichens [10]. Recognizing the roles of bacteria in fungal niches, research attention initially focused on the mycorrhizosphere. Since the pioneering work of Maria Cengia-Sambo in the 1920s [11], a number of bacteria have been isolated from cultured lichen thalli or, more recently, been detected by shotgun DNA sequencing [12,13]. Lichen-associated bacteria have also been proved to be an important contributor to lichen symbiosis [14,15,16]. A growing number of studies of the bacterial associations of lichens have changed the emphasis of research from basic taxonomic description to more in-depth functional analyses using next-generation sequencing and multi-omics approaches [14,17,18]. Among the lichen-associated bacteria, *Alphaproteobacteria* is probably the most prominent bacterial class detected, with abundant bacterial taxa representing the phyla *Acidobacteriota*, *Actinomyceota* and the *Bacteroidota*-*Chlorobiota* group (also known as *Sphingobacteria*) [18,19,20,21,22,23] (phylum names following the latest validation [24]).

The contributions bacteria make to the lichen symbiosis range across stress resistance, nitrogen fixation, provision of vitamins and acting as cofactors in the degradation of phenolic compounds [24,25]. The current study set out to analyze the lichen-associated bacterial diversity of selected Antarctic lichens by a culture-independent analysis of operational taxonomic units (OTUs) combined with OTU-derived prediction of bacterial metabolic potential in the lichen symbiosis. Rock tripe lichens (*Umbilicariaceae*) were targeted in this study because of their widespread occurrence on fellfield rocks in Antarctica, facilitating a first biogeographic comparison of OTU diversities and similarities/differences in metabolic potential in Antarctica. Lichens from the biogeographically distinct continental Antarctic and maritime Antarctic regions, sampled from Antarctic Conservation Biogeographic Regions (ACBRs) 5 and 2, respectively [26,27], were chosen for this purpose.

## 2. Materials and Methods

### 2.1. Collection of Rock Tripe Lichen Samples

**Figure 1:**
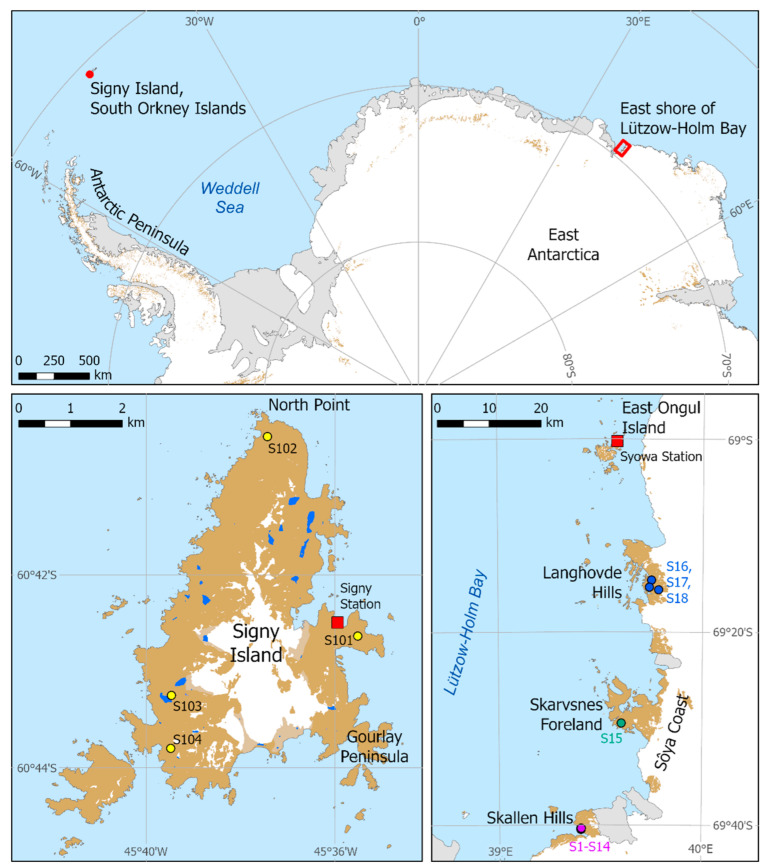
Locations of sampling sites of rock tripe lichen specimens on Signy Island (South Orkney Islands, maritime Antarctic) and the east shore of Lützow-Holm Bay, coastal Queen Maud Land, continental Antarctica. Upper panel, overview of the locations of the two sampling regions. Lower left, detailed map of Signy Island. Lower right, detailed map of the east shore of Lützow-Holm Bay near Syowa Station, showing three areas of sample collection; further details of sampling sites in the Skallen Hills are listed in Table 1.

Rock tripe lichens growing on fellfield rocks were sampled from two distinct Antarctic biogeographic regions: continental Antarctica (near Syowa Station in ACBR 5 Enderby Land) and maritime Antarctica (Signy Island in ACBR 2 South Orkney Islands) [27]. The former location was on the east shore of Lützow-Holm Bay, coastal Queen Maud Land, East Antarctica, where lichens were sampled in January and February 2011 during the 52nd Japanese Antarctic Research Expedition. On Signy Island, lichens were collected in January and February 2017 during a field season supported by the British Antarctic Survey. In total, 22 lichen samples were collected, 18 from three areas within a 50 km range in the Lützow-Holm Bay region near Syowa Station, and four from Signy Island (Figure 1, Table 1). Signy Island is about 3780 km distant from Syowa Station as calculated by Great Circle Calculator [28].

Thalli of rock tripe lichens were collected using a flame-sterilized surgical blade and tweezers and placed in pre-sterilized Whirl-Pak bags (Nasco, Fort Atkinson, WI, USA) or pre-sterilized 50 mL centrifuge tubes (As One, Osaka, Japan). The amounts of collected thalli varied between the individual sampling sites. The collected thalli were air-dried on site, stored in the dark during transportation and kept frozen at −25 °C in the laboratory until bulk DNA extraction.

### 2.2. Bulk DNA Extraction from Lichen Thalli

Approximately 1 g of lichen thalli from each sampling site was washed using autoclaved Milli-Q ultrapure water from Direct-Q UV 5 (Merck Millipore, Burlington, MA, USA). After washing, the lichen thalli were cut into small pieces using flame-sterilized scissors and ground using an autoclaved mortar and pestle. Bulk DNA was extracted from the ground thalli by bead-beating using the ISOIL Large for Beads ver.2 (Nippon Gene, Tokyo, Japan) and precipitated in 70% ethanol with the precipitation-facilitator Ethachinmate (Nippon Gene, Tokyo, Japan) [29]. The DNA precipitate was resuspended in sterilized ultrapure water, assessed for purity and quantity using a NanoDrop 2000c (Thermo Fisher Scientific, Waltham, MA, USA) and stored at −20 °C until PCR amplification.

### 2.3. Amplification and Sequencing of Fungal/Algal 18S rRNA Gene

The bulk DNA samples were used for PCR amplification of near-full-length 18S rRNA gene sequences of fungi and algae. The primers listed in Table 2 were used to amplify the target sequences by PCR using a TaKaRa PCR Thermal Cycler Dice Touch TP350 and a TaKaRa PCR Thermal Cycler PERSONAL (TaKaRa Bio, Kusatsu, Japan).

Thermal cycling for amplification of fungal 18S rRNA genes consisted of one cycle of initial denaturation at 95 °C for 5 min followed by 30 cycles of 95 °C for 45 s, 61 °C for 45 s and 72 °C for 80 s and one cycle of final elongation at 72 °C for 12 min. The same thermal cycling protocol, but with annealing at 53 °C, was used for the amplification of algal 18S rRNA genes. The eukaryotic positive controls were *Saccharomyces*-derived and *Zooxanthella*-derived DNAs. The negative control used autoclaved Milli-Q ultrapure water.

The PCR amplicons of fungal/algal 18S rRNA genes were purified by High Pure PCR Product Purification Kit (Roche, Basel, Switzerland) and Sanger-sequenced on an ABI 3730XL automatic DNA Sequencer (Thermo Fisher Scientific, Waltham, MA, USA) with BigDye Terminator v3.1 Cycle Sequencing Kit (Thermo Fisher Scientific) at the Department of Gene Science, Natural Science Center for Basic Research and Development (N-BARD), Hiroshima University [29].

### 2.4. Amplification and Sequencing of V3-V4 Region of Bacterial 16S rRNA Gene

The extracted DNA from the lichen samples was used for PCR amplification with the V3-V4 specific primers 341 F and 806 R (Table 2) using the Kapa HiFi HotStart ReadyMix PCR kit (Kapa Biosystems, Inc., Wilmington, DE, USA) on a TaKaRa PCR Thermal Cycler Dice Touch TP350 (TaKaRa Bio). The thermal cycling protocol followed was: 95 °C for 3 min, 25 cycles of 95 °C for 30 s, 55 °C for 30 s, and 72 °C for 30 s and a final elongation at 72 °C for 5 min. The sequence library was constructed following [29]. Paired-end 300 bp sequencing by MiSeq (Illumina, San Diego, CA, USA) was performed using a Nextera XT Index Kit (Illumina) at the Department of Biomedical Science, N-BARD, Hiroshima University and at the molecular diagnostic company SolGent (Daejeon, Korea).

### 2.5. Sequence Data Analysis and OTU Determination

The Sanger-generated sequences of the 18S rRNA genes were aligned by ClustalW [35] using the BioEdit sequence alignment editor [36] to remove low-quality sequences. The remaining sequences were assembled manually and Chimera-checked by tree topology analysis [37]. The resulting sequences were BLAST-searched to identify the fungal and algal partners of the studied lichens.

The MiSeq-generated V3-V4 reads were processed with the Microbiome Taxonomic Profiling (MTP) pipeline of the EzBioCloud (https://www.ezbiocloud.net/contents/16smtp; accessed on 9 February 2022) [38]. Briefly, the paired-end read merging as well as adapter and primer trimming were conducted using the EzBioCloud in-house scripts; in this step, unmerged reads, as well as ambiguous reads with <100 nucleotides or low average quality scores (<25), were omitted. For the quality-checked reads, the identical sequences were dereplicated, and then the non-redundant reads obtained were compared to the EzBioCloud 16S rRNA gene sequence database PKSSU4.0, with the option of the target taxon “bacteria”. In this PKSSU4.0 database, the uncultured taxonomic group is tentatively given the hierarchical name assigned to the DDBJ/ENA/GenBank sequence accession number with the following suffixes: “_s” (for species), “_g” (genus), “_f” (family), “_o” (order), “_c” (class) and “_p” (phylum). The taxonomic assignment was performed based on the following sequence similarity cut-offs: ≥97% for species, 97 > x ≥ 94.5% for genus; 94.5 > x ≥ 86.5% for family; 86.5 > x ≥ 82% for order; 82 > x ≥ 78.5% for class; and 78.5 > x ≥ 75% for phylum, where x corresponds to a sequence′s similarity to reference sequences [39]; reads below these cut-off values at the species or higher level were appended with the suffix “_uc” (for unclassified). All reads that could not be identified at the species level (<97% similarity) were subjected to chimera sequence detection through comparison with the EzBioCloud chimera-free reference database (https://help.ezbiocloud.net/mtp-pipeline/; accessed on 9 February 2022), and any chimera reads identified were discarded. Next, singleton reads as well as eukaryotic plastid reads were excluded. Finally, retrieved V3-V4 sequences were clustered into operational taxonomic units (OTUs) at a 97% identity cutoff value [38], which shows better universality over proposals for >98% [40]. The representative OTUs in the final data set were BLAST-searched.

The MiSeq-generated V3-V4 sequence datasets are available at DDBJ/ENA/GenBank under the DDBJ Sequence Read Archive (DRA) accession numbers DRA008580 and DRA014252, the BioProject numbers PRJDB8443 and PRJDB13657, and the BioSample numbers SAMD00175323, SAMD00175324, SAMD00175326–SAMD00175328 and SAMD00494392–SAMD00494408. The Sanger-generated 18S rRNA gene sequences were deposited in the DDBJ/ENA/GenBank database under the accession numbers LC487917, LC487919–LC487922 and LC712411–LC712427 for fungal 18S rRNA gene sequences and LC487925 and LC713009–LC713029 for algal 18S rRNA gene sequences. The sample-to-number correspondences are listed in Tables S1–S3. Note that the data from DRA008580 were obtained in our previous study [29] and were used for comparison purposes (the relevant BioProject, BioSample, and 18S rRNA gene sequence numbers are as listed above).

### 2.6. Diversity Indices and Bioinformatic Analyses of OTUs

Using the EzBioCloud MTP pipeline, rarefaction curve analysis was computed. The alpha-diversity indices (Chao1, Shannon and Simpson indices) were also calculated by the EzBioCloud MTP to estimate the richness/evenness of bacterial OTUs associated with the lichen samples (note that the Chao1 index takes singletons into account).

The beta-diversity was illustrated by principal component analysis (PCA) and hierarchical clustering based on the UniFrac distance matrix for the Antarctic OTUs [41]. Biomarker OTUs that discriminate the lichen OTU populations were specified by the linear discriminant analysis (LDA) [42] and LDA-Effect Size algorithm (LEfSe; http://huttenhower.sph.harvard.edu/galaxy/; accessed on 10 May 2022) [43]. While previous studies of lichen-associated microorganisms set the threshold LDA score to 2 [44,45], this study set the threshold to 4 and 5 in order to focus on biomarkers having large statistical differences between the two sampling regions. Differential abundance analysis was performed using the Analysis of Compositions of Microbiomes with Bias Correction (ANCOM-BC) [46].

To predict metabolic features of lichen-associated bacteria assigned from the two sampling regions, OTUs were projected on known human metabolic pathways available at Kyoto Encyclopedia of Genes and Genomes (KEGG; http://www.genome.jp/kegg/; accessed on 10 May 2022) [47] with the Visualization and Analysis of Networks containing Experimental Data (https://www.cls.uni-konstanz.de/software/vanted/; accessed on 10 May 2022) [48] and the Phylogenetic Investigation of Communities by Reconstruction of Unobserved States 2.0 (PICRUSt 2.0; https://huttenhower.sph.harvard.edu/picrust/; accessed on 10 May 2022) [49]. The bioinformatics analyses were performed using OmicStudio online tools (https://www.omicstudio.cn/tool; accessed on 10 May 2022).

## 3. Results

### 3.1. Identification of Rock Tripe Lichen-Forming Fungi and Algae

BLAST search of the near-full-length 18S rRNA gene sequences obtained showed that the fungal members of the lichens sampled in the Syowa Station region were most closely related to the ascomycetes *Umbilicaria decussata* (17 of the 18 samples) and *U. rhizinata* (one sample from the Skallen Hills). In the four samples obtained from Signy Island, three were most closely related to *U. rhizinata* and one to *U. aprina*. Similarity values were 94.2% or higher (Table S2). Intra-specific variations in the gene sequences were observed.

The algal sequences obtained were most closely related to the green algal genus *Trebouxia*, which is the most common photobiont in lichens [50], with similarity values of 98.5% or higher (Table S3). *Trebouxia aggregata* and *Trebouxia* sp. SAG 2463, which are most closely related to each other, were the most closely related cultured species to the algal partners of the studied *Umbilicaria* lichens.

### 3.2. Evaluation of MiSeq-Generated V3-V4 Sequences and OTUs

MiSeq sequencing generated a total of 1,357,573 raw reads from the 22 lichen samples, yielding 1,028,426 valid reads to be grouped into OTUs. The mean length of the valid reads was 412 bp, which is within the quantile range of Q1 403 bp and Q3 427 bp, based on the EzBioCloud database [35]. The numbers of OTUs and OTU-derived species, genera, families, orders, classes and phyla in each sample are shown in Table 3.

Table 4 shows the regional distributions of the numbers of taxa (OTU, species, genus, family, order, class and phylum) that were detected only in the samples from the Syowa Station region, only in those from Signy Island and in both regions. Higher percentages of region-specific OTUs were seen at the OTU and species ranks, while more than half of the classes and phyla were commonly seen in both regions, indicating bi-regional similarity at higher ranks and regional uniqueness at lower ranks.

Rarefaction curves were drawn based on the numbers of reads and OTUs (Figure S1). The rarefaction analysis indicated that the coverage (the ratio of an observed OTU number against an estimated OTU number; equivalent to the alpha diversity index, Chao1) ranged from 80.93% in S8 to 98.38% in S16, with an overall average of 90.43% (respective coverages can be calculated from Table 5). Therefore, the numbers of MiSeq-generated reads are considered sufficient to perform further statistical and bioinformatic analyses.

### 3.3. Taxonomic Composition of Lichen-Associated Bacterial Community

**Figure 2:**
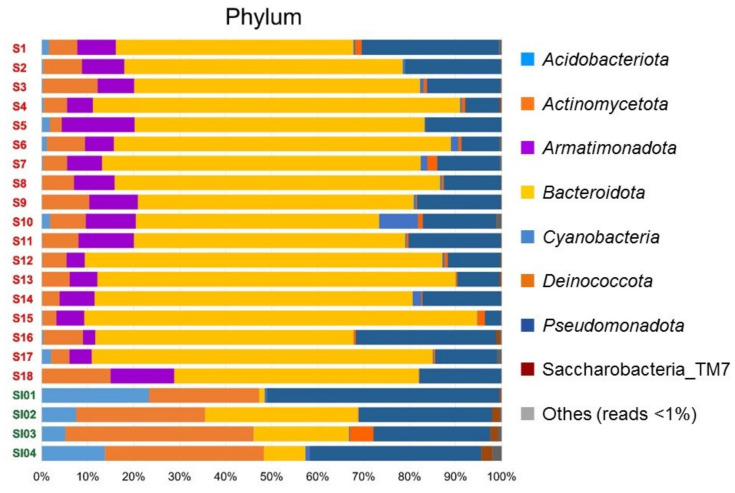
Bacterial phylum compositions of OTUs obtained from lichen samples from the Syowa Station region in continental Antarctica (S1 to S18) and Signy Island in maritime Antarctica (SI01 to SI04). Eight phyla were observed with read abundances of >1% of the total read number. Compositions of bacterial classes, orders, families, genera and species are shown in Figures S2–S5.

The compositions of the OTU-derived bacterial phyla from the 22 lichen samples are shown in Figure 2. Eight bacterial phyla were commonly present in all samples, with read abundances of >1% of total reads. Each lichen sample harbored a total 12 to 18 bacterial phyla (Table 3), with the additional 4 to 10 phyla representing <1% of reads. The common phyla were *Acidobacteriota*, *Actinomycota* (formerly *Actinobacteria*), *Armatimonadota*, *Bacteroidota*, *Cyanobacteria*, *Deinococcota*, *Pseudomonadota* (formerly *Proteobacteria*) and the candidate phylum Saccharibacteria_TM7.

### 3.4. Alpha and Beta Diversity

Alpha-diversity indices were calculated to evaluate the OTU richness of each lichen sample (Table 5). The Chao1 index values were used as the estimated OTU numbers for the rarefaction analysis. The values of the Shannon and Simpson indices were used to calculate the effective number of species (ENS) [51]. Higher Chao1, Shannon and ENS values, as well as observed OTU numbers, indicating higher species richness were found in samples from Signy Island. Lower Simpson index values calculated for Signy Island samples also indicate higher species richness and evenness.

The ENS values based on Shannon and Simpson indices were much smaller than the Chao1 values (estimated OTU numbers) as well as the observed OTU numbers and the OTU-derived species numbers (Table 3). Bacterial species richness of a large sample size may be better represented by Chao1, as also reported in other studies [52,53].

**Figure 3:**
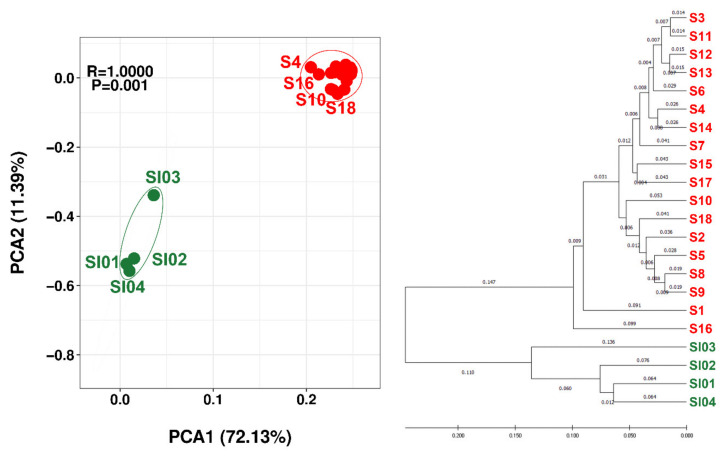
PCA plot (**left**) and hierarchical clustering dendrogram (**right**) of OTU-derived bacterial species obtained from lichens samples obtained on Signy Island (green) and the Syowa Station region (red). PCA plots at the genus, family, order, class and phylum ranks are shown in Figure S6.

Beta diversity was assessed using PCA and hierarchical cluster analysis to depict similarity/dissimilarity between samples (Figure 3). Both the PCA plot and hierarchical clustering dendrogram showed the clear separation between OTU diversity obtained from Signy Island and the Syowa Station region at the species level. Clear regional separation was also apparent at the genus, family, order, class and phylum ranks (Figure S6). In contrast, the different sampling areas within the Syowa Station region were not distinct at any taxonomic rank (PCA plots at species, genus, family, order, class and phylum rank are shown in Figure S7).

**Figure 4:**
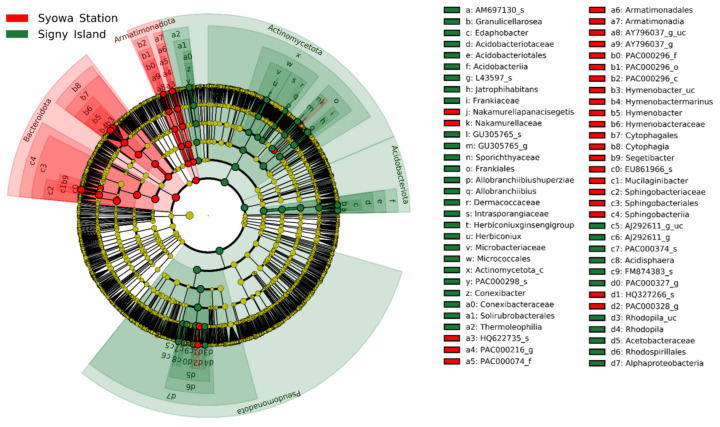
LEfSe cladogram showing taxonomic biomarkers of bacteria associated with the lichens collected from Signy Island (green) and the Syowa Station region (red). The innermost node corresponds to the domain Bacteria, followed by concentrically arranged nodes of phylum, class, order, family, genus and species. Red and green nodes/shades indicate taxa that are significantly higher in relative abundance. Diameter of each node circle is proportional to abundance of the taxon.

The regional distinction in OTU diversity was driven by biomarker OTUs or biomarker taxa, which were identified by LEfSe and are represented in the phylogenetic cladogram (Figure 4). Among these, significant biomarkers having LDA scores >5 are listed in Table 6. Significant biomarkers in the Syowa Station region included OTU EU861966_s and the derived taxa (genus to phylum) of the genus *Mucilaginibacter*, the family *Sphingobacteriaceae*, the order *Sphingobacteriales*, the class *Sphingobacteria* and the phylum *Bacteroidota*. Significant biomarkers for Signy Island were the OTU Actinomycetota_c that was affiliated with the class-level taxon within the phylum *Actinomycetota*, as well as the family *Acetobacteraceae* and its superior taxa, i.e., the order *Rhodospirillales*, the class *Alphaproteobacteria* and the phylum *Pseudomonadota*.

**Figure 5:**
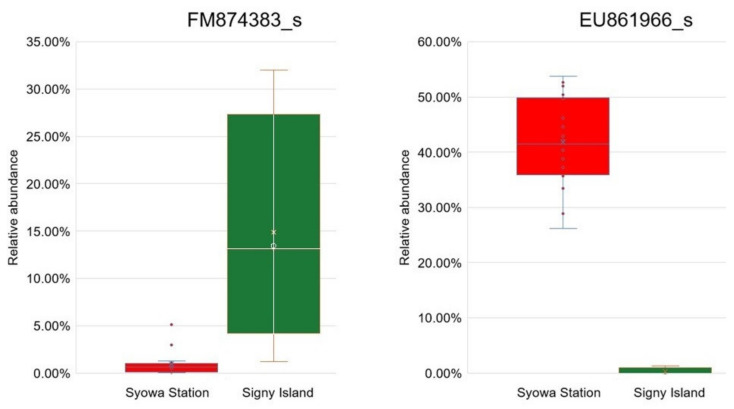
Significant differences (*p* < 0.05) in relative abundances of the top biomarker OTUs from the Syowa Station region (red) and Signy Island (green) analyzed by ANCOM-BC. (**Left**), the most significant biomarker from Signy Island, FM874383_s, affiliated with the genus *Gluconacetobacter*. (**Right**), the most significant biomarker in the Syowa Station region, EU861966_s, affiliated with the genus *Mucilaginibacter*. Other significant biomarker OTUs are shown in Figure S8.

At the species rank, only one biomarker OTU, EU861966_s from the Syowa Station region, was identified when the LDA score threshold was set to 5 (Table 6). By lowering the threshold to 4, a total of 16 biomarker OTUs (nine from Signy Island and seven from the Syowa Station region) were identified at the species rank and used for differential analysis by ANCOM-BC; the top biomarkers from the two regions are shown in Figure 5, and other biomarkers are shown in Figure S8.

**Figure 6:**
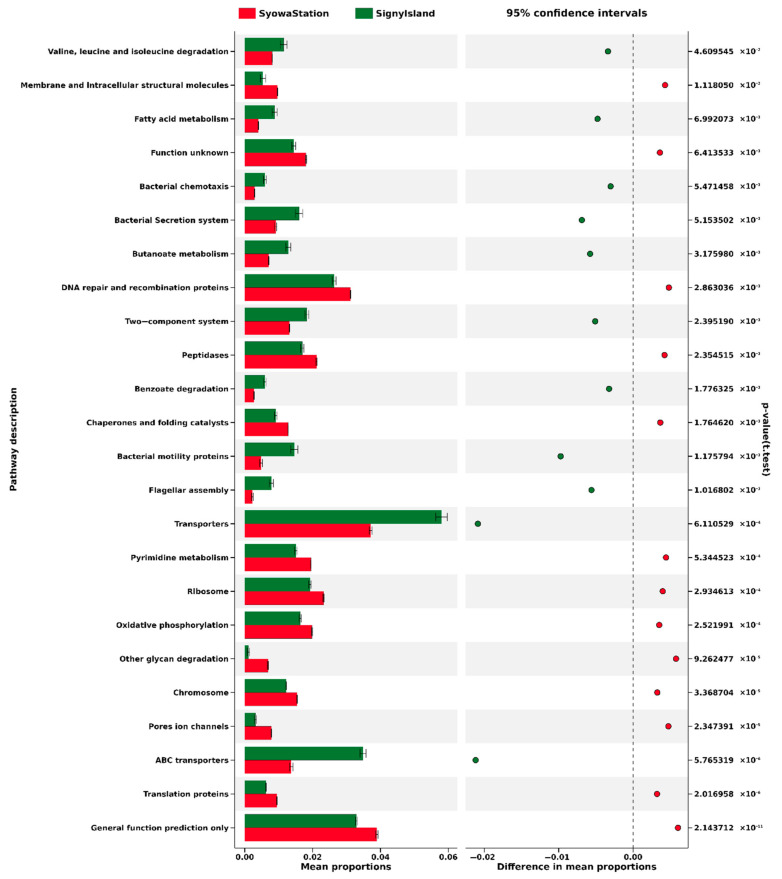
Excerpt of KEGG Level 3 metabolic pathways found in the biomarker OTUs from Signy Island (green) and the Syowa Station region (red). Horizontal axis indicates relative abundance (%) of each pathway to be compared between the two regions. The pathways are selected by cutoff value of mean relative abundance distance of two regions > |0.003|. Details of the KO functions are shown in Figure S11.

Biomarkers with LDA scores >4, including the 16 species-rank biomarkers, were further characterized by PICRUSt to predict metabolic features of lichen-associated bacteria in both sampling regions. The biomarker OTUs were projected on the LEGG metabolic map, and key metabolic features were visualized. At the KEGG Level 1, consisting of the largest metabolic categories, each OTU population showed seven large pathways, including metabolism, genetic information processing, unclassified, environmental information processing, and cellular processes, in order of relative abundance (Figure 6). Relative abundances of OTUs relevant to the “metabolism” pathway were as high as >50% in OTUs from both regions. At Level 2, consisting of sub-categories, OTUs from both sampling regions had the 26 pathways (Figure S9), of which the top five were carbohydrate metabolism (11.2% in Signy and 11.0% in Syowa), amino acid metabolism (11.0% in Signy and 10.9% in Syowa), replication and repair (7.4% in Signy and 9.2% in Syowa), energy metabolism (5.7% in Signy and 6.4% in Syowa) and membrane transport (11.3% in Signy and 6.5% in Syowa). The greatest difference in the metabolic pathways was apparent in “membrane transport”, which was more dominant in the Signy Island OTUs. At Level 3 (Figure S10), large differences were apparent in “transporters” (5.5% in Signy and 3.6% in Syowa), “ABC transporters” (3.3% in Signy and 1.3% in Syowa) and “bacterial motility proteins” (1.5% in Signy and 0.5% in Syowa), suggesting higher activities in membrane transport-mediated metabolism and cellular mobility in the Signy Island OTUs. In addition, low but higher percentages of “cell motility” and “xenobiotic biodegradation and metabolism” in Signy Island may be associated with liquid water availability due to the island’s warmer climate and potential human impact, respectively.

## 4. Discussion

Rock tripe lichens with mycobionts of the genus *Umbilicaria* and photobionts of the genus *Trebouxia* are commonly seen [29,54]. Variations in 18S rRNA gene sequences were found for *U. decussata* and *U. rhizinata* in this study, as reported in other *Umbilicaria* species [55]. The associated bacterial diversity showed no lichen species-specific grouping. For example, the bacterial OTUs associated with *U. rhizinata* sampled in the Syowa Station region and on Signy Island (samples S5 and SI01, respectively) were distinct in both PCA and hierarchical clustering analysis (Figure 3). Even comparing samples from the same general area (Skallen Hills and Langhovde Hills), bacterial OTUs associated with the same lichen species, *U. decussata*, were scattered rather than clustered on the dendrogram (Figure 3). Rather, bacterial OTUs were clearly clustered by geographical region, irrespective of the different *Umbilicaria* species. We conclude that it is not the difference in lichen species but rather the geographical/environmental setting that is the major influence on the bacterial floras associated with the *Umbilicaria* species studied here.

The influence of the algal species, *Trebouxia aggregata* and *Trebouxia* sp. SAG 2463, on the OTU floras, should not necessarily be considered. The 18S rRNA gene sequence (MT901379) of the most closely related *T. aggregata* is from the strain *T. aggregata* SAG 219-1d, isolated from the foliose lichen of the ascomycete *Xanthoria* in Europe [56] and maintained at the Culture Collection of Algae at Göttingen University (Sammlung von Algenkulturen der Universität Göttingen, SAG), Germany. The strain *Trebouxia* sp. SAG 2463 may have also been cultured at SAG but is not listed in the current SAG catalog (https://uni-goettingen.de/en/45175.html; accessed on 17 May 2022). The 18S rRNA gene sequences of the two strains are so closely related (99.8% similarity) that they would have been grouped into a single species. Therefore, we conclude that the algal photobionts of the studied *Umbilicaria* lichens should be regarded as monospecific despite intraspecific variations in 18S rRNA gene sequences being present and, thus, not influencing the composition of associated OTUs.

The number of valid reads from Signy Island was only about 10% of the total obtained, contrasting with 57% from the Syowa Station region, resulting in lower numbers being recorded at all taxonomic ranks on Signy Island. However, the assigned OTUs from Signy Island showed higher alpha-diversity, despite the smaller sample size, than those from the Syowa Station region (Table 5). In terms of beta-diversity, the Signy Island and Syowa Station regions were clearly separated by the PCA and hierarchical cluster analysis (Figure 3), while the 18 samples from three areas within a 50 km range in the Syowa Station region did not show any evidence supporting clustering of associated bacterial diversity by local area. This suggests that the ~4000 km distance between the two sampling regions is sufficient to provide a strong biogeographic barrier, while the 50 km local separation within the near-Syowa Station region is not. The possibility that the 6-year difference in sampling seasons at the two locations (2011 and 2017) may have influenced the data obtained cannot be excluded, but we consider this to be unlikely.

The difference in the maritime and continental Antarctic settings of the two sampling regions may also affect beta diversity. On Signy Island, the recorded annual mean/maximum/minimum ground temperatures are −1.7/18.4/−8.7 °C [57]. It should be noted that a (then) record high air temperature (19.8 °C) for weather stations south of 60° was reported from Signy Island in 1982 [58]. In the Langhovde area near Syowa Station, recorded annual mean/maximum/minimum ground temperatures are −8.9/21.7/−32.8 °C [59], showing lower mean and minimum ground temperatures and a wider temperature range in the continental area than the maritime region. Thus, temperature may be a key driver amongst the environmental variables likely to influence both lichen and associated bacterial occurrence and community interactions.

Higher metabolic activities predicted for the Signy Island OTUs could be related to the generally warmer and less stressful climate of this Maritime Antarctic island. Metabolism generally scales with temperature regardless of acclimation and evolutionary adaptation [60]. A warmer (and also damper) climate may favor bacterial species having higher metabolic potential, as suggested by the PICRUSt prediction (Figure 6 and Figures S9 and S10), thus influencing microbiome structures as represented by OTU diversity and biomarker OTUs. Note that the accuracy of metabolic predictions by the PICRUSt depends on the presence of reference genomes that are phylogenetically related to the OTUs detected. In order to more deeply elucidate their metabolic characteristics, future studies will need to isolate and characterize bacteria related to key OTUs or reconstruct their genomes by culture-independent metagenomic approaches (also known as metagenome-assembled genomes). The metabolism of the other lichen components, fungi and algae/cyanobacteria, also scales with temperature and may itself have direct or indirect influences on associated bacteria. Patterns of bacterial and fungal/algal metabolic interactions may vary among lichens and be subject to selection. The OTU analyses presented here provide clues to understanding the functional roles of bacteria as a third component of the lichen symbiosis, though more data are clearly required in this very young research area.

## Figures and Tables

**Table 1:** Sampling sites of rock tripe lichens along the east shore of Lützow-Holm Bay, coastal Queen Maud Land, continental Antarctica and Signy Island, maritime Antarctica. Geographical coordinates and elevations were determined with GPSMAP62S and GPSMAP76S (Garmin, Olathe, Kansas, USA), except elevations indicated with asterisks, which were inferred from topographic maps.

Region	Area	Latitude	Longitude	Elevation (m)	Sample Code
Syowa Station region: East shore of Lützow-Holm Bay, coastal Queen Maud Land, continental Antarctica	Skallen Hills	69°40′23″ S	39°24′18″ E	25	S1, S2, S3
		69°40′22″ S	39°24′11″ E	26	S4
		69°40′28″ S	39°24′14″ E	10	S5
		69°40′29″ S	39°24′12″ E	18	S6
		69°40′24″ S	39°24′10″ E	14	S7, S8, S9
		69°40′23″ S	39°24′10″ E	26	S10
		69°40′22″ S	39°24′20″ E	22	S11, S12, S13, S14
	Skarvsnes Foreland	69°29′27″ S	39°36′10″ E	* 80	S15
	Langhovde Hills	69°14′39″ S	39°44′59″ E	210	S16
		69°15′23″ S	39°44′21″ E	* 150	S17
		69°15′38″ S	39°47′04″ E	* 100	S18
Northern maritime Antarctic region	Signy Island	60°42′38″ S	45°35′31″ W	232	SI01
		60°40′34″ S	45°37′26″ W	42	SI02
		60°43′15″ S	45°39′28″ W	33	SI03
		60°43′48″ S	45°39′29″ W	79	SI04

**Table 2:** List of forward (F) and reverse (R) primers for PCR amplification of the target sequences.

Target Sequence	Primer Designation	F/R	Length (-mer)	5′ → 3′	Expected Product Size	Ref.
Fungal 18S rRNA gene	NS17UCB	F	19	CATGTCTAAGTTTAAGCAA	2.0 kbp	[30]
	NS24UCB	R	20	AAACCTTGTTACGACTTTTA		
Algal 18S rRNA gene	Euk F	F	21	AACCTGGTTGATCCTGCCAGT	1.8 kbp	[31]
	Al1700r *	R	18	CTCCTTCCTCTAGGTGGG		[32]
V3-V4 region of 16S rRNA gene	341F	F	17	CCTACGGGNGGCWGCAG	460 bp	[33]
	806R	R	21	GACTACHVGGGTATCTAATCC		[34]

* Reverse-complement of Al1700f.

**Table 3:** Numbers of MiSeq-generated V3-V4 region reads, 97% similarity-based OTUs, OTU-derived species, genera, families, orders, classes and phyla in each sample. The sub-total and total numbers of taxa are smaller than the simple sums due to overlaps among samples. Mean lengths of valid reads are also listed. Samples S1 to S18 were collected in the region of Syowa Station, and samples SI01 to SI04 from Signy Island.

Sample	Raw Read	Valid Read	OTU	Species	Genus	Family	Order	Class	Phylum	Mean Length (bp)
S1	67,130	56,304	653	499	254	109	70	43	16	411.1
S2	74,880	26,662	252	201	134	68	44	32	12	407.8
S3	74,986	63,288	482	360	193	98	63	39	16	412.3
S4	43,044	37,863	418	301	146	79	50	32	13	416.9
S5	67,503	58,877	266	203	108	66	42	29	15	410.1
S6	55,681	49,192	632	459	233	107	71	42	18	416.4
S7	58,369	50,920	489	354	178	82	51	32	13	415.0
S8	58,613	37,564	331	259	135	66	47	30	14	411.7
S9	81,518	42,341	318	249	141	76	50	33	16	408.7
S10	66,828	43,171	710	520	279	128	81	49	18	409.6
S11	73,470	63,721	319	243	128	67	42	28	15	411.0
S12	82,096	78,754	384	274	159	82	50	34	17	416.0
S13	64,184	57,538	269	204	119	71	49	31	15	416.2
S14	72,824	55,695	364	269	136	72	49	29	13	413.2
S15	51,605	38,471	332	227	134	78	50	31	15	416.3
S16	100,000	84,351	1212	392	230	107	68	38	18	413.5
S17	77,514	53,736	599	442	240	104	61	38	15	413.8
S18	77,614	34,540	193	159	93	63	47	32	14	409.2
Sub-total	1,247,859	932,988	2257	1370	623	244	129	72	25	412.7
SI01	31,714	28,693	401	278	150	74	51	32	16	405.5
SI02	33,691	29,667	553	302	162	76	45	29	14	410.7
SI03	29,792	24,259	714	490	234	96	59	36	15	411.4
SI04	14,517	12,819	617	476	233	110	63	36	18	407.6
Sub-total	109,714	95,438	1290	809	369	150	82	47	20	408.8
Total	1,357,573	1,028,426	3147	1829	762	286	144	79	27	412.0

**Table 4:** Numbers of assigned OTUs and OTU-derived species, genera, families, orders, classes and phyla that were detected only from the Syowa Station region, only from Signy Island and from both regions. The total numbers are equal to those in Table 3.

Distribution	Observed OTU	Species	Genus	Family	Order	Class	Phylum
Only in the Syowa Station region	1857	1020	393	136	62	32	7
Only in the Signy Island region	890	459	139	42	15	7	2
Common to both regions	400	350	230	108	67	40	18
Total	3147	1829	762	286	144	79	27

**Table 5:** Alpha-diversity indices (Chao1, Shannon and Simpson indices) for the bacterial OTUs assigned from 18 lichen samples from the Syowa Station region in continental Antarctica (S1–S18) and four samples from Signy Island in maritime Antarctica (SI01–SI04). Effective numbers of species (*ENS*) values were calculated from the Shannon and Simpson indices.

Sample	Observed OTU	Chao1	Shannon *(ENS)*		Simpson *(ENS)*	
S1	653	733.8	3.15	*23.3*	0.16	*6.3*
S2	252	298.1	2.14	*8.5*	0.29	*3.5*
S3	482	526.8	2.44	*11.5*	0.19	*5.3*
S4	418	448.9	2.49	*12.1*	0.21	*4.8*
S5	266	297.5	2.11	*8.3*	0.24	*4.2*
S6	632	723.3	2.66	*14.3*	0.24	*4.2*
S7	489	547.1	2.70	*14.9*	0.22	*4.6*
S8	331	408.7	2.28	*9.8*	0.29	*3.5*
S9	318	360.4	2.22	*9.2*	0.27	*3.7*
S10	710	804.7	3.48	*32.5*	0.10	*10.0*
S11	319	347.9	2.39	*10.9*	0.19	*5.3*
S12	384	418.5	2.35	*10.5*	0.22	*4.6*
S13	269	307.7	1.91	*6.8*	0.32	*3.1*
S14	364	389.7	2.47	*11.8*	0.20	*5.0*
S15	332	348.3	1.99	*7.3*	0.30	*3.3*
S16	1212	1232.5	2.55	*12.8*	0.25	*4.0*
S17	599	652.0	3.05	*21.1*	0.16	*6.3*
S18	193	218.5	2.27	*9.7*	0.20	*5.0*
**Average**	**456.8**	**503.6**	**2.48**	***13.1***	**0.23**	***4.8***
SI01	401	429.7	2.92	*18.5*	0.15	*6.7*
SI02	553	593.6	2.94	*18.9*	0.14	*7.1*
SI03	714	756.4	4.43	*83.9*	0.03	*33.3*
SI04	617	690.5	4.41	*82.3*	0.04	*25.0*
**Average**	**571.3**	**617.6**	**3.68**	***50.9***	**0.09**	***18.0***

**Table 6:** Biomarker OTUs and taxa having LDA scores >5 identified from the assigned OTU diversity obtained in the Syowa Station region and from Signy Island.

Region	Code in Figure 4	Rank of Biomarker						LDA Score	*p*-Value
		Phylum	Class	Order	Family	Genus	Species		
Syowa Station	-	*Bacteroidota*						5.46	0.002
	c4	*Bacteroidota*	*Sphingobacteria*					5.34	0.002
	c3	*Bacteroidota*	*Sphingobacteria*	*Sphingobacteriales*				5.34	0.002
	c2	*Bacteroidota*	*Sphingobacteria*	*Sphingobacteriales*	*Sphingobacteriaceae*			5.34	0.002
	c1	*Bacteroidota*	*Sphingobacteria*	*Sphingobacteriales*	*Sphingobacteriaceae*	*Mucilaginibacter*		5.34	0.002
	c0	*Bacteroidota*	*Sphingobacteria*	*Sphingobacteriales*	*Sphingobacteriaceae*	*Mucilaginibacter*	EU861966_s	5.32	0.002
Signy Island	-	*Actinomycetota*						5.13	0.002
	y	*Actinomycetota*	Actinomycetota_c					5.03	0.002
	-	*Pseudomonadota*						5.07	0.004
	d7	*Pseudomonadota*	*Alphaproteobacteria*					5.07	0.004
	d6	*Pseudomonadota*	*Alphaproteobacteria*	*Rhodospirillales*				5.09	0.005
	d5	*Pseudomonadota*	*Alphaproteobacteria*	*Rhodospirillales*	*Acetobacteraceae*			5.07	0.005

## Data Availability

The raw sequence data, project data and sample data are available at the DDBJ Sequence Read Archive (DRA008580 and DRA014252), BioProject (PRJDB8443 and PRJDB13657), and BioSample (SAMD00175323, SAMD00175324, SAMD00175325–SAMD00175328 and SAMD00494392–SAMD00494408), respectively.

## Supplementary Materials

The following supporting information can be downloaded at: https://www.mdpi.com/article/10.3390/jof8080817/s1. Table S1: List of BioProject numbers, DDBJ Sequence Read Archive (DRA) accession numbers, and BioSample accession numbers of the V3-V4 sequence datasets registered to the public database of DDBJ; Table S2: List of the accession numbers of near-full-length fungal 18S rRNA gene sequences of the studied rock tripe lichen samples, the accession numbers and the most closely related species with respective accession numbers, as well as similarity values (%); Table S3: List of the accession numbers of near-full-length algal 18S rRNA gene sequences of the studied rock tripe lichen samples, the accession numbers and the most closely related species with respective accession numbers, as well as similarity values (%). Figure S1: Rarefaction curves based on the numbers of reads and OTUs from 18 sites in the Syowa Station region and 4 sites on Signy Island; Figure S2: Bacterial class compositions of OTUs in lichen samples from the Syowa Station region in East Antarctica (S1 to S18) and Signy Island in the Maritime Antarctic (SI01 to SI04); Figure S3: Bacterial order compositions of OTUs in lichen samples from the Syowa Station region (S1 to S18) and Signy Island in the Maritime Antarctic (SI01 to SI04); Figure S4: Bacterial family compositions of OTUs in lichen samples from the Syowa Station region (S1 to S18) and Signy Island in the Maritime Antarctic (SI01 to SI04); Figure S5: Bacterial genus compositions of OTUs in lichen samples from the Syowa Station region (S1 to S18) and Signy Island in the Maritime Antarctic (SI01 to SI04); Figure S6: PCA plots of OTU-derived species (upper left), genera (upper middle), families (upper right), orders (lower left), classes (lower middle) and phyla (lower right) of the lichens sampled from Signy Island (green) and the Syowa Station region (red); Figure S7: PCA plot of OTU-derived species of the lichens sampled from three areas (purple, thin blue and yellow in Figure 1) within the Syowa Station region; Figure S8: Significant differences (*p* < 0.05) in relative abundances of biomarker OTUs from the Syowa Station region (red) and Signy Island (green) analyzed by ANCOM-BC. Names of possibly affiliated genera or phyla are shown below the OTU names if applicable; Figure S9: KEGG Level 2 metabolic pathways found in the biomarker OTUs from Signy Island (green) and the Syowa Station region (red); Figure S10: KEGG Level 3 metabolic pathways found in the biomarker OTUs from Signy Island (green) and the Syowa Station region (red). Figure S11. Excerpt of KO functions found in the biomarker OTUs from Signy Island (green) and the Syowa Station region (red). Horizontal axis indicates relative abundance (%) of each function to be compared between the two regions. The functions are selected by cut-off value of mean relative abundance distance of two region > |0.0015|.
